# Concurrent Structural and Functional Patterns in Patients With Amnestic Mild Cognitive Impairment

**DOI:** 10.3389/fnagi.2022.838161

**Published:** 2022-05-17

**Authors:** Li Liu, Tenglong Wang, Xiangdong Du, Xiaobin Zhang, Chuang Xue, Yu Ma, Dong Wang

**Affiliations:** ^1^Affiliated Mental Health Center, Hangzhou Seventh People’s Hospital, Zhejiang University School of Medicine, Hangzhou, China; ^2^School of Humanities and Management, Graduate School of Wannan Medical College, Wuhu, China; ^3^Department of Geriatric Psychiatry, Suzhou Mental Health Center, Suzhou Guangji Hospital, The Affiliated Guangji Hospital of Soochow University, Suzhou, China

**Keywords:** amnestic mild cognitive impairment, voxel-based morphometry, amplitude of low-frequency fluctuations, regional homogeneity, resting-state functional connectivity

## Abstract

Amnestic mild cognitive impairment (aMCI) is a clinical subtype of MCI, which is known to have a high risk of developing Alzheimer’s disease (AD). Although neuroimaging studies have reported brain abnormalities in patients with aMCI, concurrent structural and functional patterns in patients with aMCI were still unclear. In this study, we combined voxel-based morphometry (VBM), amplitude of low-frequency fluctuations (ALFFs), regional homogeneity (Reho), and resting-state functional connectivity (RSFC) approaches to explore concurrent structural and functional alterations in patients with aMCI. We found that, compared with healthy controls (HCs), both ALFF and Reho were decreased in the right superior frontal gyrus (SFG_R) and right middle frontal gyrus (MFG_R) of patients with aMCI, and both gray matter volume (GMV) and Reho were decreased in the left inferior frontal gyrus (IFG_L) of patients with aMCI. Furthermore, we took these overlapping clusters from VBM, ALFF, and Reho analyses as seed regions to analyze RSFC. We found that, compared with HCs, patients with aMCI had decreased RSFC between SFG_R and the right temporal lobe (subgyral) (TL_R), the MFG_R seed and left superior temporal gyrus (STG_L), left inferior parietal lobule (IPL_L), and right anterior cingulate cortex (ACC_R), the IFG_L seed and left precentral gyrus (PRG_L), left cingulate gyrus (CG_L), and IPL_L. These findings highlighted shared imaging features in structural and functional magnetic resonance imaging (MRI), suggesting that SFG_R, MFG_R, and IFG_L may play a major role in the pathophysiology of aMCI, which might be useful to better understand the underlying neural mechanisms of aMCI and AD.

## Introduction

Mild cognitive impairment (MCI) is an early but abnormal state of cognitive impairment, which is considered a transitional period between normal aging and early Alzheimer’s disease (AD) (Petersen, 2010), usually characterized by cognitive decline, and without dementia (Petersen et al., 2009). According to the difference in the impaired cognitive domain, there are two major types of MCI: amnestic MCI (aMCI) and non-amnestic MCI (naMCI) (Petersen et al., 2014). aMCI is characterized by memory deficits and, to a large extent, often leads to AD. Actually, people with aMCI have a high risk of developing AD, and about 10–15% of patients with aMCI will progress to AD, while the annual rate in the normal population is 1–2% (Petersen et al., 2001). AD has become a social problem in recent decades due to its heavy financial burden and poor effective treatment. However, the pathophysiology of AD and aMCI remains unclear.

Neuroimaging studies may provide valuable information to predict the incidence and development of aMCI and have great potential to provide the pathological process that leads to cognitive decline. Recently, numerous studies have reported damage to structural or functional changes in the brain of patients with aMCI. Structural magnetic resonance imaging (sMRI) studies have shown the changes of gray matter (GM) atrophy in many regions such as in the amygdala, hippocampus (HP), medial temporal lobe, and thalamus in aMCI (Nickl-Jockschat et al., 2012; Zhang J. et al., 2021). Resting-state functional MRI (rs-fMRI) is a supplement to sMRI, which can describe functional changes in the whole brain (Zhang et al., 2020). The impairment of functional brain activity occurred mainly in the default mode network (DMN), executive control network (ECN), and salience network (SN) in aMCI (Li et al., 2020; Fu et al., 2021; Xue et al., 2021a). Liu et al. (2021) suggested that the impairment of functional brain activity occurred mainly in the DMN and language network in MCI. However, the results of these structural and functional MRI studies were inconsistent and difficult to replicate. Therefore, the combination of functional and structural analysis may provide new insights into an understanding of the changes in the brain of patients with aMCI.

In recent years, several studies have used combined structural and functional MRI in patients with aMCI. Some studies focused on specific predefined brain networks or regions (such as the DMN, SN, or HP) (Bharath et al., 2017; Wang et al., 2020; Xue et al., 2021b), or focused on specific band oscillations (Zhao et al., 2015) to investigate the difference between patients with aMCI and the other groups. Some studies have used machine learning methods to investigate structural and functional patterns between patients with aMCI and the other groups (Wee et al., 2012; Yan et al., 2019). However, the results of these studies were inconsistent due to small samples or inconsistent parameters. Especially, these studies also did not describe concurrent structural and functional connectivity patterns in aMCI. Therefore, in this study, we aimed to combine voxel-based morphometry (VBM), amplitude of low-frequency fluctuations (ALFFs), regional homogeneity (Reho), and seed-based resting-state functional connectivity (RSFC) to explore possible concurrent structural and functional changes in patients with aMCI. We hypothesized that patients with aMCI have concurrent functional and structural brain regions and that these regions may play an important role in aMCI.

## Materials and Methods

### Participants

The study was conducted under the ethical approval of the Ethics Committee of Suzhou Guangji Hospital, and all individuals gave written informed consent prior to participation. A total of 232 subjects were recruited in this study from July 2019 to March 2021, including 122 patients with aMCI and 110 healthy controls (HCs). Patients with aMCI were screened to meet the Peterson MCI criteria (Petersen et al., 1999): (1) had a memory complaint; (2) Mini-Mental State Examination (MMSE) scores between 24 and 30; (3) objective memory loss adjusted for education and age; (4) a Clinical Dementia Rating (CDR) of 0.5; (5) normal or near-normal performance in cognitive function without significant levels of impairment in other cognitive domains; (6) the absence of dementia according to Diagnostic and Statistical Manual of Mental Disorders, 4th edition, revised (DSM-IV); and (7) essentially preserved activities of daily living. HCs were enrolled as described in the structured interview for DSM-IV non-patient edition to confirm the lifelong absence of psychiatric and neurological illness. Exclusion criteria applied to all subjects were as follows: mental and neurological diseases, history of stroke, substance abuse, several medical conditions that cause cognitive impairment, such as syphilis, thyroid dysfunction, severe anemia, and HIV.

### Magnetic Resonance Imaging Data Acquisition

All data were acquired with the GE Discovery MR750W 3.0 T System (General Electric Discovery silent, United States) at the Suzhou Guangji Hospital. Functional imaging data (echo-planar imaging, EPI sequence) were obtained with the following parameters: repetition time = 2000 ms; echo time = 30 ms; flip angle = 90°; field of view (FOV) = 224 mm × 224 mm; acquisition matrix = 64 × 64; 36 slices; 200 volumes; voxel size = 3.5 × 3.5 × 3.5; and slice thickness = 3.6 mm. Structural imaging data were collected (3D T1-weighted SFPGR sequence) with the following parameters: repetition time = 7.7 ms; echo time = 3.1 ms; FOV = 256 mm × 256 mm; and voxel size = 1 mm × 1 mm × 1 mm. The scan time lasts for 400 s. All subjects were asked to keep their eyes closed and remain awake during the scan.

### Data Analysis

#### Clinical Data Analysis

Demographic and clinical variables were analyzed with SPSS25.0 (IBM, IL, United States). Data with non-normality were log-transformed into a normal distribution. Two-sample *t*-tests were used to compare differences in age, education, and MMSE scores between the two groups. χ^2^-test was used to compare gender differences between the two groups. *p* < 0.05 was statistically significant.

#### Structural Magnetic Resonance Imaging Analysis

Voxel-based morphometry data were processed with the VBM8 tool of the SPM8 software package^1^ on the MATLAB R2012a platform (The MathWorks, Natick, MA, United States). First, T1 images were visually inspected for anomalies by orienting them to place the anterior commissure at the origin of the Montreal Neurological Institute (MNI) 3D coordinate system. Then, the images were normalized to template space and segmented into GM, white matter (WM), and cerebrospinal fluid (CSF) using SPM8 standard unified segmentation. The next step was spatial normalization of the segmented GM and WM images using the DARTEL algorithm (Ashburner, 2007). Finally, the normalized GM images were smoothed by a Gaussian kernel with full width at half maximum (FWHM) of 8 mm. A voxel-wise analysis with two-sample *t*-tests was conducted to detect an abnormality in gray matter volume (GMV) between the aMCI group and HC group with age, sex, years of education, and total intracranial volume (TIV) as covariates. Correction for multiple comparisons was performed with *p* < 0.01 [false discovery rate (FDR) correction for multiple comparisons].

#### Resting-State Functional MRI Analysis

The rs-fMRI data preprocessing was carried out with SPM8 and DPABI V4.3.^2^ The data were processed as follows: (1) The first 10 volumes were discarded to reduce scan noise and magnetic field instability. (2) Slice timing and head motion in the rs-fMRI images were corrected. (3) Coregistered, segmentation, and regression of the nuisance signals of the WM signal, CSF signal, and head motion parameters. (4) The data were normalized to the MNI space and resampled to a voxel size of 3 mm × 3 mm × 3 mm. (5) Frames with a displacement (FD) greater than 0.5 mm were removed. (6) Detrended, bandpass filtering from 0.01 to 0.08 Hz was carried out in Reho analysis, and smoothing with an 8-mm FWHM Gaussian kernel was carried out in the ALFF analysis.

#### Amplitude of Low-Frequency Fluctuation and Regional Homogeneity Analyses

We compared the Reho and ALFF differences between aMCI and the HC group in SPM8 and DPABI V4.3. The detailed Reho measurement was described in our previous research (Liu et al., 2021). Briefly, individual Reho maps were performed by calculating Kendall’s coefficient concordance (KCC) of the time series of a given voxel with its neighboring 26 voxels (Zang et al., 2004). Then, the data were smoothed with an 8-mm FWHM Gaussian kernel to generate Reho maps for each subject in each group. Fast Fourier transform (FFT) was used to transform the filtered time series to the frequency domain to obtain the power spectrum. Then, the square root was calculated at each frequency of the power spectrum and the root mean square at 0.01–0.08 Hz was obtained for each voxel as ALFF values. Subsequently, similar to Reho analysis, the ALFF value of each voxel was divided by the global mean ALFF value within the whole-brain mask (Yang et al., 2007). The significance of group differences was set at *p* < 0.01 using the FDR correction for multiple comparisons, accompanied with age, gender, and years of education as covariates.

#### Seed-Based Resting-State Functional Connectivity Analysis

To further characterize the nature of RSFC alterations in aMCI, whole-brain analyses restricted to overlapping brain regions that were repeatedly reported in previous findings were conducted. These important clusters that showed an significant brain region overlap during VBM, ALFF, and Reho analyses were selected as the seed regions of interest (ROIs). In this study, we selected the peak coordinates of the left inferior frontal gyrus (IFG_L), right superior frontal gyrus (SFG_R), and right middle frontal gyrus (MFG_R) to create spherical regions with a radius of 5 mm as ROIs. Then, we extracted the average time series of each ROI and calculated the Pearson correlation between the time series of whole-brain voxels and each ROI to generate the FC maps for each subject. Subsequently, the *z*-map was obtained using Fisher’s *z* transformation to improve normality. Finally, we compared the global connectivity difference of the three ROIs between the two groups using two-sample *t*-tests. The significance of group differences was set at *p* < 0.01 using the FDR correction with age, gender, and years of education as covariates.

## Results

### Baseline Characteristics

A total of 232 subjects were recruited in this study. A total of 17 subjects were excluded due to excessive movement and direction during the scan. Finally, 114 patients with aMCI and 101 HCs were included in the next sMRI and rs-fMRI analysis. Demographic and clinical data are shown in Table 1. There were no significant differences between patients with aMCI and the HC group in terms of gender (χ^2^ = 0.50, *p* = 0.49), age (*F* = 0.78, *p* = 0.44), and education (*F* = 1.21, *p* = 0.23). Additionally, compared with the HC group, patients with aMCI had significantly lower MMSE scores (*F* = −33.85, *p* < 0.001).

**TABLE 1:** Demographic and clinical characteristics of patients with aMCI and HC.

	aMCI	HC	*F*/χ^2^ values	*p*-Values
Number	114	101		
Female/male	68/46	65/36	0.50	0.49^a^
Age (years)	72.35 ± 5.23	71.69 ± 4.95	0.78	0.44^b^
Formal education (years)	10.78 ± 3.71	10.24 ± 2.73	1.21	0.23^b^
MMSE score	24.11 ± 1.01	28.31 ± 0.97	−33.85	<0.001^b^
CDR score	0.5	0		

*aMCI, amnesic mild cognitive impairment; HC, healthy control; MMSE, Mini-Mental State Examination; CDR, Clinical Dementia Rating; values are mean ± standard deviation (SD).*

*^a^The value of p was obtained by using the χ^2^ test.*

*^b^The p-value was obtained using two-sample t-tests.*

### Voxel-Based Morphometry, Amplitude of Low-Frequency Fluctuation, and Regional Homogeneity Differences Between Amnestic Mild Cognitive Impairment and Healthy Control

#### Voxel-Based Morphometry Results

**FIGURE 1:**
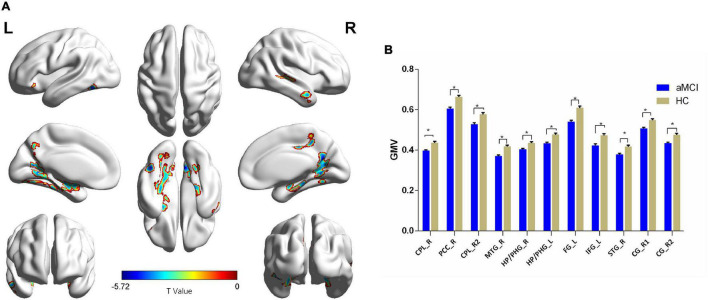
The VBM comparison between patients with aMCI and the HC group. **(A)** Significant clusters obtained from two-sample *t*-tests, the color bar represents the range of *t*-values; **(B)** brain regions showing decreased gray matter volume (GMV) in patients with aMCI. The error bars represent the standard error of the mean (SEM) and asterisks show significant differences between the groups; a threshold of *p* < 0.01, false discovery rate (FDR) correction, only clusters with *k* = 100 or large are mentioned. CPL, cerebellum posterior lobe; PCC, posterior cingulate cortex; MTG, middle temporal gyrus; HP, hippocampus; PHG, parahippocampal gyrus; FG, fusiform gyrus; IFG, inferior frontal gyrus; STG, superior temporal gyrus; CG, cingulate gyrus; aMCI, amnesic mild cognitive impairment; HC, healthy control; VBM, voxel-based morphometry; L, left; R, right. *p < 0.01.

Compared with the HC group, patients with aMCI showed significantly decreased GMV in the right cerebellum posterior lobe (CPL_R), right posterior cingulate cortex (PCC_R), right middle temporal gyrus (MTG_R), bilateral HP, and bilateral parahippocampal gyrus (PHG), left fusiform gyrus (FG_L), IFG_L, right superior temporal gyrus (STG_R), and right cingulate gyrus (CG_R) (*p* < 0.01, FDR corrected). Additionally, compared with the HC group, patients with aMCI showed no significantly increased volumes in any brain region (Table 2 and Figure 1).

**TABLE 2:** The VBM, ALFF, and Reho comparisons between patients with aMCI and HCs.

Contrast	Brain regions	Voxels	Brodmann areas	Peak MNI (*X*, *Y*, *Z*)			*Z* score
**VBM comparison between aMCI and HC**							
aMCI < HC	R cerebellum posterior lobe	697	NA	25.5	−70.5	−39	−4.39
	R posterior cingulate cortex	6470	18/19/30/31	7.5	−60	22.5	−5.42
	R cerebellum posterior lobe	163	NA	4.5	−64.5	−36	−4.60
	R middle temporal gyrus	220	20/21	48	0	−22.5	−5.51
	R parahippocampal gyrus/hippocampus	370	35/36	21	−31.5	−12	−4.42
	L parahippocampal gyrus/hippocampus	938	28/34/35	−31.5	−18	−13.5	−4.75
	L fusiform gyrus	224	37	−40.5	−60	−13.5	−5.72
	**L inferior frontal gyrus**	**102**	**47**	**−46.5**	**21**	**−1.5**	**−4.40**
	R superior temporal gyrus	161	22	52.5	−18	−3	−4.14
	R cingulate gyrus	346	5/7/31	1.5	−33	43.5	−4.56
	R cingulate gyrus	141	19/24	9	−24	37.5	−4.81
aMCI > HC	No brain region above the threshold						
**ALFF comparison between aMCI and HC**							
aMCI < HC	L thalamus	268	NA	−15	−21	0	−4.86
	L anterior cingulate cortex	979	24	−9	21	24	−5.53
	L precentral gyrus	190	9/44	−42	0	27	−4.18
	**R superior/middle frontal gyrus**	**511**	**6/40**	**24**	**12**	**42**	**−5.03**
aMCI > HC	No brain region above the threshold						
**Reho comparison between aMCI and HC**							
aMCI < HC	L posterior cingulate cortex	1162	24/23	−3	−30	24	−5.65
	R inferior frontal gyrus	140	NA	42	27	15	−5.74
	**R superior/middle frontal gyrus**	**1337**	**6/40**	**24**	**12**	**42**	**−5.86**
	**L inferior frontal gyrus**	**166**	6/44	**−39**	**3**	**27**	**−5.00**
aMCI > HC	No brain region above the threshold						

*aMCI, amnesic mild cognitive impairment; HC, healthy control; L, left; R, right; NA, not applicable; MNI, Montreal Neurological Institute; X, Y, Z, indicate the coordinates according to the MNI; VBM, voxel-based morphometry; ALFFs, amplitude of low-frequency fluctuations; Reho, regional homogeneity.*

*A threshold of p < 0.01, false discovery rate (FDR) correction, only clusters with k = 100 or larger are mentioned.*

*Bold terms and values indicating overlapping brain region.*

#### Amplitude of Low-Frequency Fluctuation Results

**FIGURE 2:**
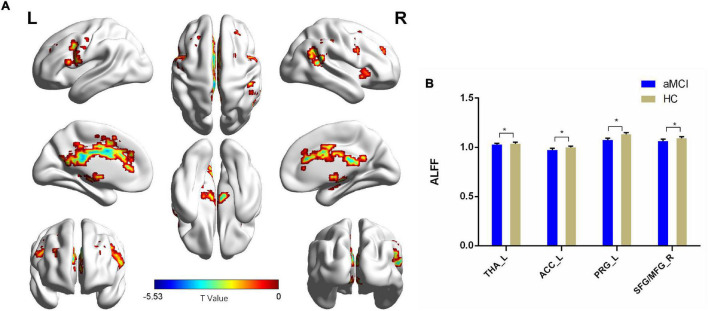
The ALFF comparison between patients with aMCI and the HC group. **(A)** Significant clusters obtained from two-sample *t*-tests, the color bar represents the range of *t*-values; **(B)** brain regions showing decreased ALFF in patients with aMCI. The error bars represent the SEM and asterisks show significant differences between the groups; a threshold of *p* < 0.01, FDR correction, only clusters with *k* = 100 or large are mentioned. THA, thalamus; ACC, anterior cingulate cortex; PRG, precentral gyrus; SFG, superior frontal gyrus; MFG, middle frontal gyrus; aMCI, amnesic-mild cognitive impairment; HC, healthy control; ALFFs, amplitude of low-frequency fluctuations; L, left; R, right. *p < 0.01.

Compared with the HC group, patients with aMCI showed decreased ALFF values in the left thalamus (THA_L), left anterior cingulate cortex (ACC_L), left precentral gyrus (PRG_L), SFG_R, and MFG_R (*p* < 0.01, FDR corrected). Additionally, compared with the HC group, patients with aMCI showed no significantly increased ALFF values in any brain region (Table 2 and Figure 2).

#### Regional Homogeneity Results

**FIGURE 3:**
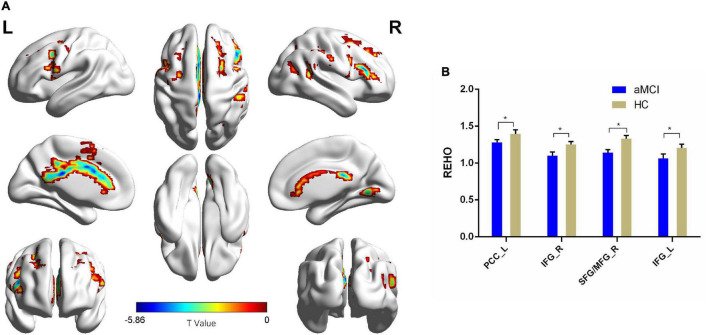
The Reho comparison between patients with aMCI and the HC group. **(A)** Significant clusters obtained from two-sample *t*-tests, the color bar represents the range of *t*-values; **(B)** brain regions showing decreased Reho in patients with aMCI. The error bars represent the SEM and asterisks show significant differences between the groups; a threshold of *p* < 0.01, FDR correction, only clusters with *k* = 100 or large are mentioned. PCC, posterior cingulate cortex; IFG, inferior frontal gyrus; SFG, superior frontal gyrus; MFG, middle frontal gyrus; aMCI, amnesic-mild cognitive impairment; HC, healthy control; Reho, regional homogeneity; L, left; R, right. *p < 0.01.

Compared with the HC group, patients with aMCI showed decreased Reho values in PCC_L, the bilateral inferior frontal gyrus (IFG), SFG_R, and MFG_R (*p* < 0.01, FDR corrected). Additionally, compared with the HC group, patients with aMCI showed no significantly increased Reho values in any brain region (Table 2 and Figure 3).

### Resting-State Functional Connectivity Differences Between Amnestic Mild Cognitive Impairment and Healthy Control

#### Right Superior Frontal Gyrus Resting-State Functional Connectivity Results

Comparing the ALFF and Reho results, only one shared cluster showed alterations in both ALFF and Reho in patients with aMCI. Considering that the size of this cluster was very large, including two key brain regions (SFG_R and MFG_R) associated with aMCI, we chose the peak coordinates of SFG_R and MFG_R as the two ROIs for the following RSFC analysis.

Using SFG_R as the ROI, the RSFC analysis revealed that FC values of the right temporal lobe (subgyral) (TL_R) were reduced in patients with aMCI than in the HC group. Additionally, compared with the HC group, patients with aMCI showed no significantly increased RSFC between SFG_R and any other brain region (Table 3).

**TABLE 3:** Regions showing seed-based functional connectivity differences between the two groups.

Seed	Brain regions	Voxels	Peak MNI (*X*, *Y*, *Z*)			*Z* score
**R superior frontal gyrus**						
aMCI < HC	R temporal lobe (subgyral)	61	42	−27	−12	−5.06
aMCI > HC	No brain regions above the threshold					
**R middle frontal gyrus**						
aMCI < HC	L superior temporal gyrus	132	−54	−3	9	−4.59
	L inferior parietal lobule	117	−39	−39	30	−4.47
	R anterior cingulate cortex	151	12	30	18	−4.86
aMCI > HC	No brain regions above the threshold					
**L inferior frontal gyrus**						
aMCI < HC	L precentral gyrus	35	−42	0	30	−4.47
	L cingulate gyrus	104	−3	12	45	−6.36
	L inferior parietal lobule	46	−42	−63	39	−4.14
aMCI > HC	No brain regions above the threshold					

*aMCI, amnesic mild cognitive impairment; HC, healthy control; L, left; R, right; MNI, Montreal Neurological Institute; X, Y, Z, indicate the coordinates according to the MNI.*

*A threshold of p < 0.01, FDR correction, only clusters with k = 35 or larger are mentioned.*

#### Right Middle Frontal Gyrus Resting-State Functional Connectivity Results

Using MFG_R as the ROI, the RSFC analysis displayed that FC values of the left superior temporal gyrus (STG_L), left inferior parietal lobule (IPL_L), and right anterior cingulate cortex (ACC_R) were reduced in patients with aMCI than in the HC group. Additionally, compared with the HC group, aMCI patients showed no significant differences of RSFC between MFG_R and any other brain region (Table 3).

#### Left Inferior Frontal Gyrus Resting-State Functional Connectivity Results

In comparison of the VBM and ALFF/Reho results, only IFG_L shared the GMV and Reho alterations in patients with aMCI. Therefore, we selected the peak coordinate of IFG_L as the ROI for the RSFC analysis.

Using IFG_L as the ROI, the RSFC analysis found that FC values of PRG_L, the left cingulate gyrus (CG_L), and IPL_L were reduced in patients with aMCI than in the HC group. Additionally, compared with the HC group, patients with aMCI had no significantly increased RSFC between IFG_L and any other brain region (Table 3).

## Discussion

In this study, large samples and multi-modal data methods were used to explore the structural and resting-state functional neuroimaging changes in patients with aMCI, and to seek for concurrent patterns of brain functional and structural changes in patients with aMCI. We found that both ALFF and Reho were decreased in the SFG_R and MFG_R of patients with aMCI, and both GMV and Reho were decreased in the IFG_L of patients with aMCI. Furthermore, we used the overlapping clusters derived from VBM, ALFF, and Reho analyses as ROIs for the RSFC analysis, which can provide reasonable and persuasive results. And, this finding showed that RSFC between the SFG_R seed and TL_R (subgyral) was decreased; RSFC between the MFG_R seed and STG_L, IPL_L, and ACC_R was decreased; and RSFC between the IFG_L seed and PRG_L, CG_L, and IPL_L was also decreased in patients with aMCI. These important results support the involvement of SFG_R, MFG_R, and IFG_L in the pathophysiology of aMCI.

### Altered Resting-State Functional Connectivity Patterns of Right Superior Frontal Gyrus in Patients With Amnestic Mild Cognitive Impairment

Amplitude of low-frequency fluctuations reflects the intensity of spontaneous brain activity (Yang et al., 2007), and Reho reflects the synchronization of spontaneous brain activity (Zang et al., 2004). In patients with aMCI, Zhang Z. et al. (2021) observed decreased Reho in the superior frontal gyrus (SFG) and middle frontal gyrus. Wang et al. (2021) observed decreased ALFF in SFG. Our study showed that the SFG_R of patients with aMCI had both decreased ALFF and Reho values, which is consistent with previous studies. Therefore, we speculated that the weakened spontaneous neuronal activity of SFG_R might help to distinguish aMCI from HC. SFG is mainly located in the upper part of the prefrontal cortex and includes multiple subregions (Li et al., 2013). SFG is a core region of the dorsolateral prefrontal cortex (DLPFC) (Koenigs and Grafman, 2009), and it plays a key role in ECN, which is associated with executive dysfunction. Evidence shows that episodic memory, executive function, language, and visuospatial function were the major impaired cognitive domains in multi-domain patients with aMCI (Winblad et al., 2004), suggesting that SFG_R may participate in executive dysfunction in patients with aMCI.

To further explore the correlation between SFG_R and other brain regions in patients with aMCI, SFG_R was chosen as the ROI for the RSFC analysis. In this study, we found that, compared with HCs, decreased RSFC in the aMCI group was mainly in TL_R.

The temporal lobe is located below the lateral sulcus of the brain, associated with hearing, memory, and emotion (Li et al., 2021). Xie et al. (2015) reported a decrease in functional connectivity in the TL in patients with aMCI. Additionally, they found that the medial TL was impaired in the early stages of AD. Along with disease progression, the damage might extend to other regions, eventually leading to cognitive impairments. According to our results, RSFC alteration was mainly found in the TL, which was consistent with the study conducted by Xie et al. (2015). Based on the abovementioned results, we inferred that abnormal functional connectivity of the TL may lead to cognition dysfunction in patients with aMCI, and the TL may be an effective biomarker in monitoring the progression of AD.

### Altered Resting-State Functional Connectivity Patterns of Right Middle Frontal Gyrus in Patients With Amnestic Mild Cognitive Impairment

The middle frontal gyrus is located mainly in the lateral prefrontal cortex, a core region of the DLPFC (Koenigs and Grafman, 2009), and it plays a key role in the ECN. It has been reported to be associated with episodic memory and emotional processing (Carballedo et al., 2011; Rajah et al., 2011). In this study, we found that the MFG_R of patients with aMCI had both decreased ALFF and Reho, which was consistent with previous studies (Wang et al., 2021; Zhang Z. et al., 2021). Based on the abovementioned findings, abnormal spontaneous activity of MFG_R may be related to executive dysfunction and episodic memory in the aMCI group.

To further explore the correlation between MFG_R and other brain regions in patients with aMCI, MFG_R was used as the ROI for the RSFC analysis. In this study, we found that, compared with HCs, decreased RSFC in the aMCI group was mainly in the STG_L, IPL_L, and ACC_R, which were functionally associated with the DMN, ECN, and auditory network.

The anterior cingulate cortex is related to cognition, emotional processing, and executive function (Fillinger et al., 2018; Jung et al., 2019). The inferior parietal lobule (IPL) is associated with episodic memory, semantic processing, and spatial cognitive function (Wang et al., 2017). Both the anterior cingulate cortex (ACC) and IPL belong to the DMN (Buckner et al., 2008; Raichle, 2015). The DMN is an important network, which is closely involved in episodic memory processing and emotion regulation in patients with cognitive decline (Raichle, 2015; Xie et al., 2016). It plays a crucial role in the progression of AD (Greicius et al., 2004). Consistent with our findings, numerous studies reported a typical disruption of the DMN in patients with AD and aMCI (Li et al., 2020; Ma et al., 2020). Actually, the deposition of β-amyloid proteins occurs in the DMN and might reduce the connection with other brain regions (Wang et al., 2013). Hence, an abnormal RSFC between the middle frontal gyrus and DMN may be related to altered cognition in patients with aMCI, which provides valuable insights into identifying high-risk groups for AD. Additionally, STG is an important region of the language network, involved in language and episodic memory (Yi et al., 2019; Liu et al., 2021). In this study, decreased RSFC in the STG_L may reflect an intimate relationship between STG_L and language dysfunction in patients with aMCI.

### Altered Resting-State Functional Connectivity Patterns of Left Inferior Frontal Gyrus in Patients With Amnestic Mild Cognitive Impairment

In this study, we found that the IFG_L of patients with aMCI had concurrent structural and functional changes, which suggested that IFG_L might be a better indicator for predicting cognitive deficits in aMCI (Gilmore et al., 2021). IFG_L was related to language/semantic processing. Xue et al. (2021b) found negative associations between IFG_L and cognitive domains in patients with aMCI, such as executive function and working memory. Therefore, we inferred that alterations in the IFG_L might be associated with executive function and the language network.

To further explore the correlation between IFG_L and the other brain regions in patients with aMCI, IFG_L was selected as the ROI for the RSFC analysis. We found that, compared to HC, decreased RSFC in the aMCI group was mainly in PRG_L, CG_L, and IPL_L, which were functionally associated with the sensorimotor network (SMN), DMN, and limbic system.

Precentral gyrus is involved in motor and executive functions, and it plays a central role in the SMN (Chenji et al., 2016; Feng et al., 2018). The SMN is mainly composed of visual, auditory, and sensory-motor cortex. Studies reported that changes in sensory and motor function may be earlier than cognitive symptoms in AD and may increase the risk of AD (Albers et al., 2015). These findings suggested that the SMN may be a predictor of conversion to AD. Additionally, Wang et al. (2015) proposed that the functional connectivity of the SMN was firstly impaired in AD and then extended to other key regions in AD, suggesting that the SMN may coordinate with other networks, and lead to clinical symptoms of patients with AD and MCI. In these data, decreased RSFC in the PRG implicated in the SMN could explain the impairment of the sensory-motor function and executive function in aMCI. As a core region of the limbic system, CG is mainly involved in the regulation of emotional state (Heimer and Hoesen, 2006). Yang et al. (2015) reported that emotional stimuli were thought to enhance episodic memory through the production of automatic attention and the old/new parietal effect. Based on this finding, we speculate that CG may affect episodic memory through emotion regulation. In addition, CG was found to play an important role in the whole-brain language network (Battistella et al., 2019). Therefore, decreased CG RSFC may be involved in multiple cognitive domains, including language and episodic memory impairments in aMCI.

### Limitations

Although these findings have been of great value, there are still several limitations. First, the present study was a cross-sectional, single-center design with a small sample size and may not have sufficient power. In the future, longitudinal and multicenter studies with large sample sizes are required to explore the relationship between structural and functional findings. Second, there was no detection of biology-related data and genetic information. Third, further patient recruitment in the prodromal and more severe stages of AD is warranted to understand the structural–functional association in the preclinical AD spectrum. Fourth, fMRI and VBM analyses using SPM in this study might give rise to the observation of false-positive functional and structural changes (Eklund et al., 2016; Gorriz et al., 2019). The data still need to be interpreted with caution.

## Conclusion

In summary, using combined structural and functional MRI analyses, we found the shared brain region alterations in patients with aMCI. SFG_R, MFG_R, and IFG_L were detected as the primary regions that may be involved in various cognitive deficits in patients with aMCI, from both structural and functional perspectives. Our results suggested that these damaged brain areas might play a major role in the aMCI stage of AD, which may help to better understand complicated neurobiology mechanisms and provide crucial insights into imaging methods for early diagnosis, intervention, and more effective prevention for MCI and AD.

## Data Availability Statement

The raw data supporting the conclusions of this article will be made available by the authors, without undue reservation.

## Ethics Statement

The studies involving human participants were reviewed and approved by the Ethics Committee of Suzhou Guangji Hospital. The patients/participants provided their written informed consent to participate in this study.

## Author Contributions

LL wrote the first draft of the manuscript. TW and YM were responsible for data collection. DW was responsible for study design, data analysis, and subsequent editing. CX, XZ, and XD made critical revisions to this manuscript. All authors contributed to the article and approved the submitted version.

## Conflict of Interest

The authors declare that the research was conducted in the absence of any commercial or financial relationships that could be construed as a potential conflict of interest.

## Publisher’s Note

All claims expressed in this article are solely those of the authors and do not necessarily represent those of their affiliated organizations, or those of the publisher, the editors and the reviewers. Any product that may be evaluated in this article, or claim that may be made by its manufacturer, is not guaranteed or endorsed by the publisher.

## Footnotes

1
https://www.fil.ion.ucl.ac.uk/spm/

2
http://www.restfmri.net/forum/DPARSF
